# Case report: A homozygous *ADAMTSL2* missense variant causes geleophysic dysplasia with high similarity to Weill-Marchesani syndrome

**DOI:** 10.3389/fgene.2022.1014188

**Published:** 2022-09-28

**Authors:** Mojiang Li, Yingshu Li, Huixing Liu, Haiyan Zhou, Wanqin Xie, Qinghua Peng

**Affiliations:** ^1^ Hunan University of Chinese Medicine, Changsha, China; ^2^ The First Affiliated Hospital of Hunan University of Chinese Medicine, Changsha, China; ^3^ Department of Vision Center, Liuyang Jili Hospital (Liuyang Eye Hospital), Changsha, China; ^4^ Department of Ophthalmology (Division II), Liuyang Jili Hospital (Liuyang Eye Hospital), Changsha, China; ^5^ National Health Committee Key Laboratory of Birth Defects for Research and Prevention, Hunan Provincial Maternal and Child Health Care Hospital, Changsha, China

**Keywords:** ADAMTSL2, geleophysic dysplasia, Weill-Marchesani syndrome, acromelic dysplasias, missense mutation, microspherophakia, lens subluxation, high myopia

## Abstract

**Background:** Geleophysic dysplasia and Weill-Marchesani syndrome from the acromelic dysplasias group of genetic skeletal disorders share remarkable clinical and genetic overlap.

**Methods:** Ophthalmological, physical, radiological examinations were conducted with a female patient in her early 30 s. Whole exome sequencing followed by Sanger sequencing validation was performed to identify the genetic cause.

**Results:** The patient, born to consanguineous Chinese parents, presented with microspherophakia, lens subluxation, high myopia, short statue, small hands and feet, stiff joints, and thickened skin. A diagnosis of Weill-Marchesani syndrome was initially made for her. However, genetic testing reveals that the patient is homozygous for the c.1966G>A (p.Gly656Ser) variant in *ADAMTSL2*, and that the patient’s healthy mother and daughter are heterozygous for the variant. As mutations in *ADAMTSL2* are known to cause autosomal recessive geleophysic dysplasia, the patient is re-diagnosed with geleophysic dysplasia in terms of her genotype and phenotype.

**Conclusion:** The present study describes the clinical phenotype of the homozygous *ADAMTSL2* p. Gly656Ser variant, which increases our understanding of the genotype-phenotype correlation in acromelic dysplasias.

## Introduction

Geleophysic dysplasia (GD, OMIM#231050, 614185, 617809) and Weill-Marchesani syndrome (WMS, OMIM#277600, 608328, 614819, 603196) are closely related conditions in the acromelic dysplasias group according to the 2019 nosology and classification of skeletal disorders (Mortier et al., 2019). Both conditions are characterized by short stature, brachydactyly, and restricted joint mobility (Pimienta et al., 2013). Apart from the common features, GD is characteristic of a round, full ‘happy’ face and severe cardiac and/or respiratory diseases leading to a high risk of early death. The distinctive features of WMS include microspherophakia with associated lens dislocation and myopia (McInerney-Leo et al., 2016).

Genetic overlap also exists between GD and WMS. Mutations in the *fibrillin1* (*FBN1*) gene can cause GD or WMS with autosomal dominant inheritance (Faivre et al., 2003; Le Goff et al., 2011). Mutations in *ADAMTSL2* (a disintegrin and metalloproteinase with thrombospondin repeats-like 2) and *LTBP3* (*latent transforming growth factor β binding protein 3*) are responsible for autosomal recessive and dominant GD, respectively (Le Goff et al., 2008; Allali et al., 2011). *LTBP2*, *ADAMTS17* and *ADAMTS10* are known to cause autosomal recessive WMS (Morales et al., 2009; Haji-Seyed-Javadi et al., 2012).

In an earlier study that linked missense mutations in *ADAMTSL2* with autosomal recessive GD, functional assays showed that ADAMTSL2 missense mutants had reduced extracellular secretion, and that ADAMTSL2 interacted with LTBP1 (latent TGF-β-binding protein 1), a protein well-known for a storage role of latent TGF-β in the extracellular matrix. Importantly, a significant increase in total and active TGF-β in the culture medium was observed for fibroblasts derived from GD patients. These findings suggest that the GD-causing mutations in *ADAMTSL2* result in a dysregulation of TGF-β signaling (Le Goff et al., 2008). The *FBN1* gene, which is previously well characterized as a causative gene for Marfan syndrome, encodes the glycoprotein fibrillin-1 that forms microfibrils in extracellular matrix and act as a TGF-β modulator (Chaudhry et al., 2007). In another study of GD case series, the dominant disease-causing mutations of *FBN1* were found to be concentrated in its TGF-β-binding protein-like domain 5 (TB5) and associated with microfibrillar network disorganization and enhanced TGF-β signaling in GD fibroblasts. Interestingly, ADAMTSL2 was found to directly interact with FBN1 in the study (Le Goff et al., 2011). The interplays of *ADAMTS* superfamily members, *FBN1* and *LTBPs* suggest that these disease-causing genes all are involved in TGF-β pathway *via* directly or indirectly regulating the bioavailability of TGF-β in extracellular matrix (Le Goff and Cormier-Daire, 2012; Przyklenk et al., 2022).

Due to the great phenotypic and genetic overlap, differentiation between GD and WMS sometimes can be difficult. The present study reports a patient who carries a homozygous missense mutation in the *ADAMTSL2* gene and displays a GD phenotype with high similarity to WMS.

## Materials and methods

### Medical ethics

The Medical Ethics Committees of Liuyang Jili Hospital (KYSZ 2022-005) and Hunan Provincial Maternal and Child Health Care Hospital approved the study. All individuals gave written informed consent to the study.

### Patient and clinical examinations

A woman in her early 30s with high myopia presented to our vision center for a corrective lens prescription. She was referred for further diagnostic evaluation. Ophthalmological, physical, radiological examinations were conducted with her.

### WES sequencing and data analysis

WES was conducted at Berry Genomics (Beijing, China). Briefly, Genomic DNA was extracted from 200 μL peripheral blood using a Qiagen DNA Blood Midi/Mini kit (Qiagen GmbH, Hilden, Germany) following the manufacturer’s protocol. For library construction, 50 ng DNA was fragmented to 200 bp around by enzymes. The DNA fragments were then end-repaired, and the 3′end was added 1 A base. Next, the DNA fragments were ligated with barcoded sequencing adaptors, and fragments of about 320 bp were collected by AMPure XP magnetic beads (Beckman Coulter, Brea, CA, United States). After PCR amplification, the DNA fragments were hybridized and captured by IDT’s xGen Exome Research Panel V1.0 (Integrated DNA Technologies, San Diego, United States) according to the manufacturer’s protocol. The hybrid products were eluted and collected, and then subjected to PCR amplification and the purification The prepared libraries were quantified by qPCR and size distribution were determined using an Agilent Bioanalyzer 2,100 (Agilent Technologies, Santa Clara, CA, United States). Finally, Novaseq6000 platform (Illumina, San Diego, United States), with 150 bp pair-end sequencing mode, was used for sequencing the genomic DNA. Raw image files were processed using CASAVA v1.82 for base calling and generating raw data. The sequencing reads were aligned to the human reference genome (hg19/GRCh37) using Burrows–Wheeler Aligner tool and PCR duplicates were removed by using Picard v1.57 (http://picard.sourceforge.net/). Verita Trekker^®^ Variants Detection System by Berry Genomics and the third-party software GATK (https://software.broadinstitute.org/gatk/) were employed for variant calling. Variant annotation and interpretation were conducted by ANNOVAR (Wang et al., 2010) and the Enliven^®^ Variants Annotation Interpretation System authorized by Berry Genomics. The human population databases, such as gnomAD (http://gnomad.broadinstitute.org/), the 1,000 Genome Project (http://browser.1000genomes.org), dbSNP (http://www.ncbi.nlm.nih.gov/snp), and internal database (Berrybig data), and the disease and phenotype databases such as OMIM (http://www.omim.org), ClinVar (http://www.ncbi.nlm.nih.gov/clinvar), and HGMD (http://www.hgmd.org) were used for annotation.

## Results

### Clinical presentation

**FIGURE 1:**
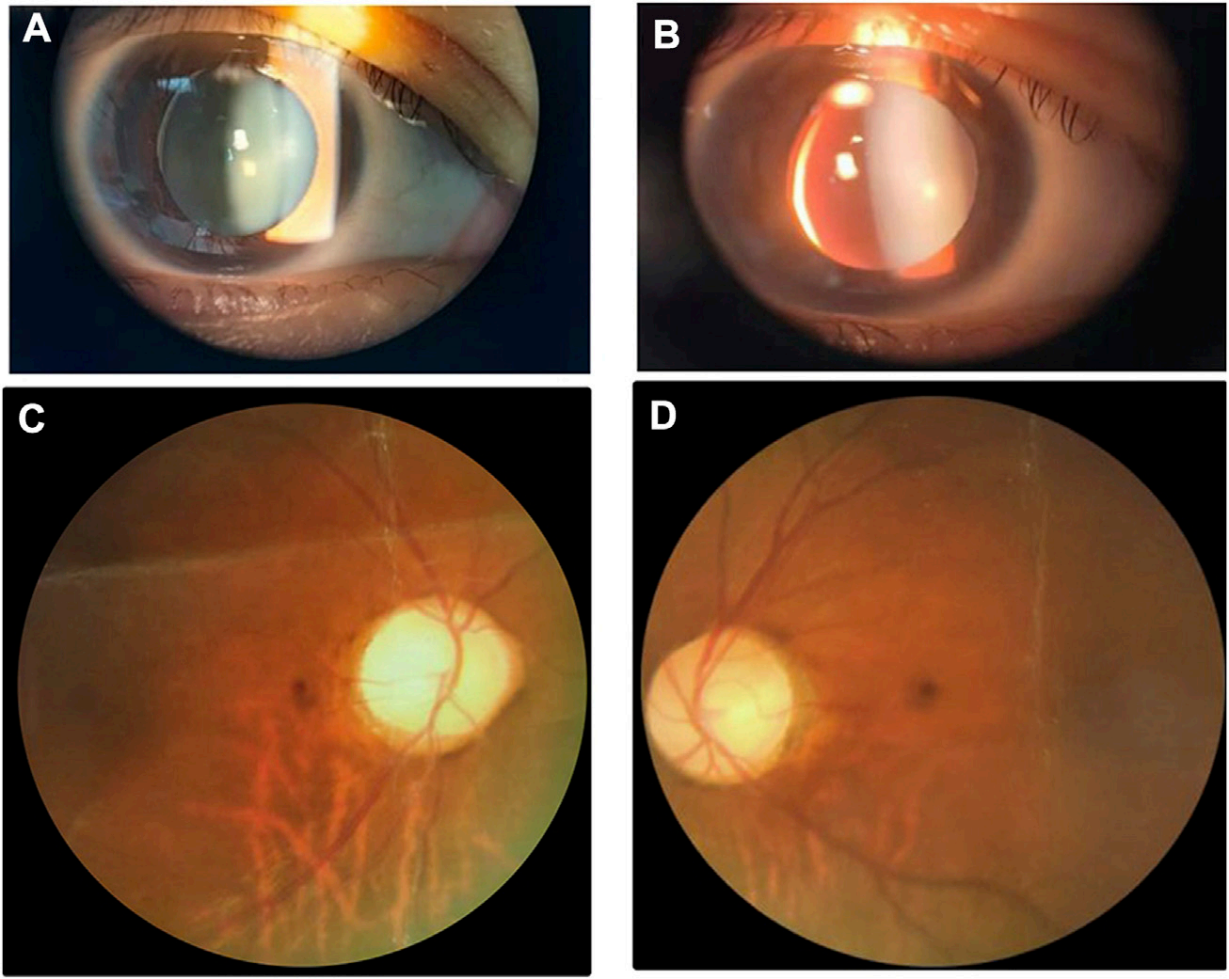
Ocular findings of the patient. Slit-Lamp images show small, clear lens with increased thickness and inferior-nasal subluxation of the right **(A)** and left **(B)** eyes. Fundus images of the right and left eyes are shown in **(C)** and **(D)**, respectively.

**FIGURE 2:**
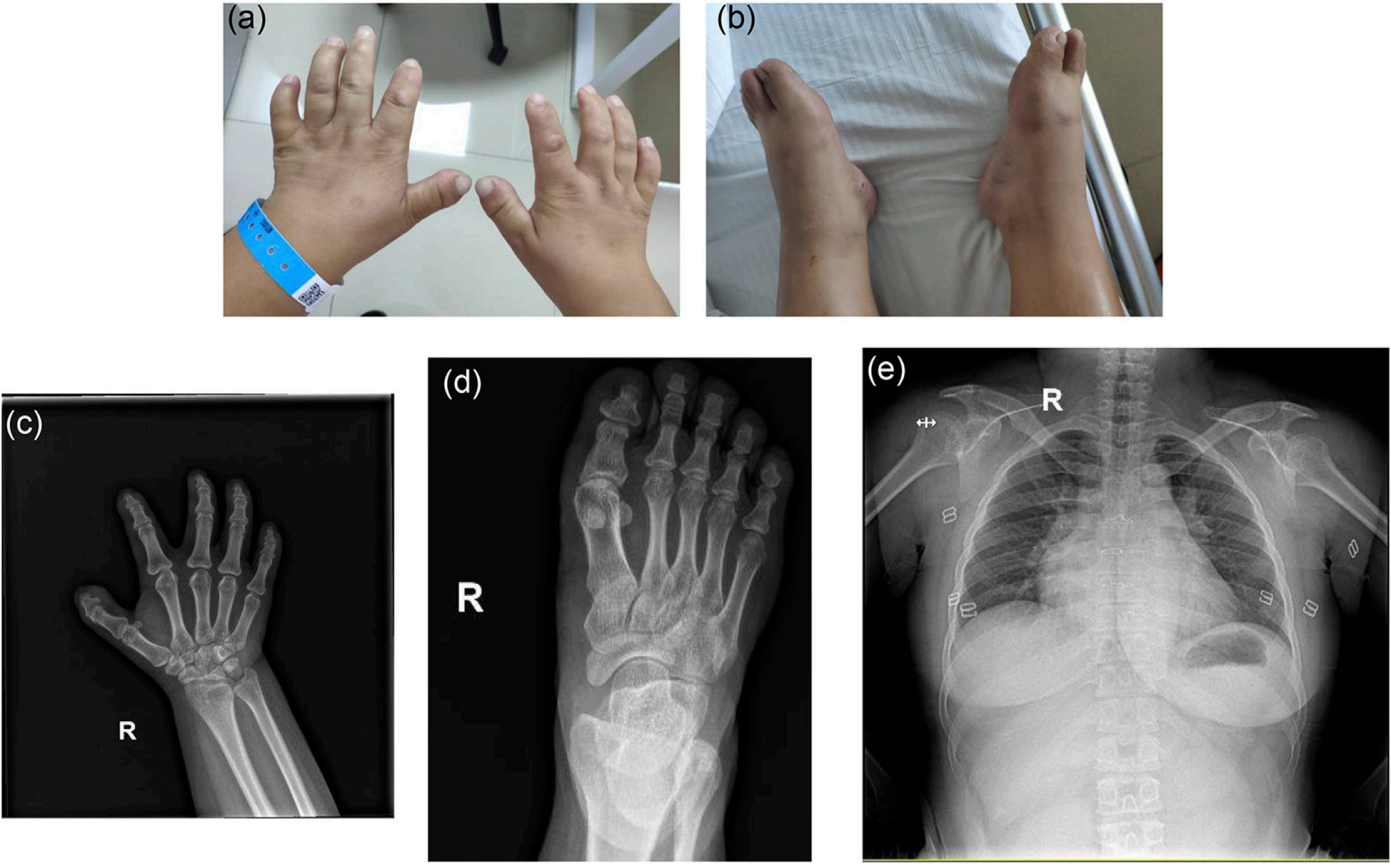
Clinical features of the patient on physic and radiographic examinations. **(A,B)** Note the short hands and feet. **(C,D)** Digital radiography reveals short tubular bones and cone shaped epiphyses in extremities (R, right). **(E)** Note the enlarged cardiac shadow and sternum closure metal wires.

**FIGURE 3:**
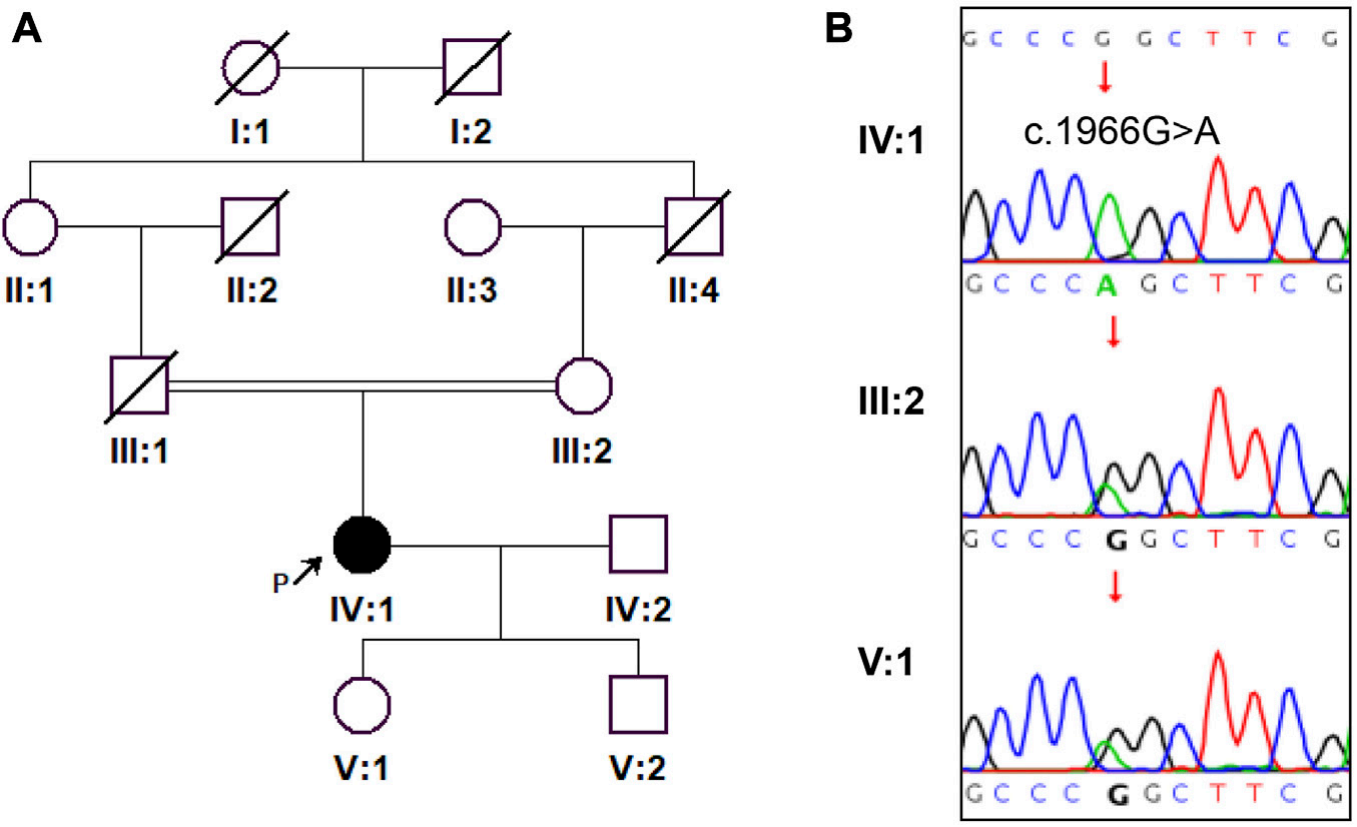
A pedigree of the family and Sanger sequencing results are shown. **(A)** The proband is indicated by a black arrow. **(B)** Sanger sequencing confirmed the homozygosity of *ADAMTSL2* c.1966G>A (p.Gly656Ser) mutation in the proband.

With respect to the patient, refractive errors as measured by computer optometry were as follows: right eye -13.50 DS/-1.25 DC × 110° and left eye -14.25 DS/-4.25 DC × 155°. Goldmann applanation intraocular pressure (IOP) was 41 mm Hg in both the left and right eyes. Slit-lamp examination revealed shallow anterior chamber, iridodonesis, and small, clear lens with increased thickness and inferior-nasal subluxation after pupillary dilation (Figures 1A,B **).** Fundus photography showed pale color of the optic papilla, cup-disc ratio of 0.5, and alteration in the fundus (Figures 1C,D). Optical coherence tomography (OCT) revealed thinning of the retinal nerve fiber layer (Supplementary Figure S1). Single field analysis revealed peripheral arcuat visual field defect at upper temporal quadrants of both eyes (Supplementary Figure S2). Ultrasound biomicroscopy (UBM) examination also suggested shallow anterior chamber, and additionally, iris pushed forward (Supplementary Figure S3). Ocular ultrasound examination showed short axial lengths (right eye 21.19 mm and left eye 21.21 mm) and no remarkable findings in the posterior segments of both eyes. On physic examination, the patient had a history of congenital heart disease and received cardiac surgery in middle childhood. She had a round face with full cheeks, upturned mouth corner, low-set ears, short statue (height 146 cm [2 SD], weight 50 kg), short neck, small hands and feet, thickened skin, stiff joints and normal intelligence (Figures 2A,B). She was born to consanguineous Chinese parents, and had mothered two children (Figure 3A). History of similar conditions in her parents and kids was denied. Serum biochemical tests were unremarkable. Digital radiography revealed short tubular bones with cone-shaped epiphysis in the feet and hands, an enlargement of cardiac shadow, and sternum closure metal wires indicative of previous surgery (Figures 2C–E). The patient was initially diagnosed as WMS based on the observed clinical symptoms, especially the ocular findings distinguishing for WMS (microspherophakia, lens subluxation, and high myopia). 0.2% bromonidine tartrate (Alphagan) and 1% brinzolamide (AZOPT) eye drops were given to relieve IOP; and the IOP was lowered to 24 mm Hg in right eye and 26 mm Hg in left eye on the third day upon hospitalization. After discharge from the hospital, Alphagan and AZOPT were prescribed for daily use. The IOP was measured bimonthly and maintained at 22∼23 mm Hg for both eyes.

### Genetic findings

To genetically confirm the clinical diagnosis, the patient and her mother and daughter volunteered for genetic testing. WES revealed a homozygous *ADAMTSL2* (NM_014694.4): c.1966G>A: p. Gly656Ser mutation in the patient. Sanger sequencing confirmed the homozygosity of the p. Gly656Ser variant in the patient, and the heterozygosity of the variant in the patient’s mother and daughter (Figure 3B). The variant has not been present in the ExAC, 1,000 Genomes or gnomAD database. The variant has been indexed in HGMD and classified as deleterious mutation (DM) as supported by two publications (Zhang et al., 2004; Piccolo et al., 2019). According to the ACMG guidelines, the variant is regarded as variant of uncertain significance (Rules PM2+PM3_supporting). Mutations in *ADAMTSL2* have been known to cause GD. Moreover, the p. Gly656Ser variant has previously been reported to cause autosomal recessive GD in compound heterozygosity with the p. Arg648Pro variant, and has a damaging effect based on *in silicon* analysis (Zhang et al., 2004; Piccolo et al., 2019). In terms of the patient’s genotype and phenotype, the patient is eventually diagnosed with GD, and the homozygous *ADAMTSL2* p. Gly656Ser mutation is considered as the genetic cause for the patient. Other genetic variants detected by WES but with insufficient evidence to support their pathogenicity were excluded for further analysis (Table 1).

**TABLE 1:** The variants with insufficient evidence to support their pathogenicity in whole exome sequencing for the family.

Gene	Chromosome location	Gene subregion	HGVS	Mutation type	MAF	Heterozygosity	ACMG grading	Diseases and inheritance
*PKDCC*	chr2:42055393-42055393	exon 5	NM_138370.3: c.1222G>A:p.E408K	nonsynonymous SNV	0.000588928	Proband: Het; Mother: WT; Daughter: WT	VUS	Rhizomelic limb shortening with dysmorphic features, OMIM#618821, AR
*P3H2*	chr3:190120473- 190120473	exon1	NM_018192.4: c.259C>T: p.H87Y	nonsynonymous SNV		Proband: Het; Mother: Het; Daughter: Het	VUS	High myopia with cataract and vitreoretinal degeneration; OMIM#614292, AR
*SPARC*	chr5:151669684- 151669684	exon6	NM_0031 18.4: c.431A>G: p.D144G	nonsynonymous SNV		Proband: Het; Mother: WT; Daughter: WT	VUS	Osteogenesis imperfect type xvii; OMIM#616507, AR
*ESC O 2*	chr8:27776690- 27776690	exon3	NM_0010 17420.3: c.382G>A: p.G128R	nonsynonymous SNV	0.00691119	Proband: Het; Mother: Het; Daughter: WT	VUS	Juberg-Hayward syndrome, OMIM#216100, AR; Roberts-SC phocomelia syndrome, OMIM#268300, AR
*SLC26A2*	chr5:149981105- 149981105	exon3	NM_000112.4: c.1512G>A: p.M504I	nonsynonymous SNV	0.00380559	Proband: Het; Mother: Het; Daughter: Het	VUS	Achondrogenesis, type ib, OMIM#600972, AR; De La Chapelle dysplasia, OMIM#256050, AR; Diastrophic Dysplasia, OMIM#222600, AR; Epiphyseal dysplasia multiple 4, OMIM#226900AR.

HGVS, human genome variation society; MAF, minor allele frequency; ACMG, american college of medical genetics and genomics; SNV, single nucleotide variation; Mother, the proband’s mother; Daughter, the proband’s daughter; Het, Heterozygous; WT, wild type; VUS, variant of uncertain significance; AR, autosomal recessive; OMIM, online mendelian inheritance in man.

## Discussion

Both geleophysic dysplasia (GD) and Weill-Marchesani syndrome (WMS) belong to the acromelic dysplasias group of genetic skeletal disorders (Mortier et al., 2019). These two conditions share remarkable clinical and genetic overlap, presenting a challenge for differential diagnosis. In a previous study by Pimienta et al., a female patient with a characteristic ‘happy’ face was diagnosed with GD in early childhood. However, a rediagnosis of WMS was made in her teens based on the genetic finding of compound heterozygous mutations in *ADAMTS10*. Of note, her GD facial features had evolved to be atypical at reevaluation (Pimienta et al., 2013). In the present study, the patient presented to our center in her 30s and typical geleophysic facial features were not observed. Her ocular features including microspherophakia, lens subluxation, and high myopia were prominent, highly suggesting a possibility of WMS. However, autosomal recessive GD is implicated by the patient’s homozygous p. Gly656Ser mutation in *ADAMTSL2*. Our study highlights a diagnostic value of genetic evidence for different forms of acromelic dysplasias.

In a previous study, a 35-year-old woman with GD caused by bi-allelic splice-site mutations in *ADAMTSL2* was reported to have microspherophakia, lens subluxation, keratoglobus, and high myopia (Kochhar et al., 2013). These ocular findings are regarded as distinctive features of WMS. Notably, the ocular phenotype of the patient in our study is similar to that of the 35-year-old woman in the previous study. The 35-year-old woman showed a less typical ‘happy’ face in adulthood than in childhood. We speculate that the patient in our study might also have an evolving phenotype of facial features and so that typical geleophysic face was not observed in her 30s. We are aware of that the ocular findings associated with compound heterozygous mutations p. Gly656Ser and p. Arg648Pro in ADAMTSL2 were previously described in a 9-year-old girl diagnosed with GD (Zhang et al., 2004). She had increased intraocular pressure (IOP), short axial length, thick and steep central corneas, thick crystalline lenses, and shallow anterior chambers. The ocular phenotype of the patient with homozygous ADAMTSL2 p. Gly656Ser mutation in our study mainly showed increased IOP, short axial length, microspherophakia, lens subluxation, high myopia, large cup-disc ratio (C/D = 0.5), optic nerve atrophy and shallow anterior chambers. The partial phenotypic overlap between the two patients (e.g. increased IOP, short axial length and shallow anterior chambers) might be explained by the genetic overlap, both patients possessing an *ADAMTSL2* p. Gly656Ser allele. The large cup-disc ratio and visual field defect of the patient in our study suggests a high risk of secondary angle-closure glaucoma. Therefore, periodic ophthalmologic examinations are warranted for the patient.

ADAMTSL2 is a 951-residue protein characterized by a signal peptide, an N-terminal thrombospondin type-1 repeat (TSR1), a cystein-rich module, a spacer module, an N-glycan-rich module, six tandem TSRs (TSR2-7), and a C-terminal PLAC (protease and lacunin) domain (Koo et al., 2007). Interestingly, six of the seven TSRs contain the consensus sequence for *O*-fucosylation modification; and GD-causing mutations within ADAMTSL2 TSRs have been recurrently observed. Recent evidence indicates that *O*-fucosylation of ADAMTSL2 is required for secretion; and disruption of fucosylation due to GD-causing mutations within ADAMTSL2 TSRs may impair the protein secretion, highlighting a potential disease mechanism (Zhang et al., 2020). The p. Gly656Ser variant of ADAMTSL2 was previously reported to cause autosomal recessive GD in compound heterozygosity with the p. Arg648Pro variant (Piccolo et al., 2019). Both the glycine residue 656 and the arginine residue 648 are localized in the TSR3 domain. *In silicon* analysis performed by Piccolo et al. show that the p. Gly656Ser mutation probably has a damaging effect on the protein structure and function (Piccolo et al., 2019). In our study, the clinical features of an adult female patient with homozygous ADAMTSL2 p. Gly656Ser mutation was first described, providing new insights into the genotype-phenotype correlation of *ADAMTSL2* mutations. It will be interesting to determine the potential fucosylation and secretion alterations of ADAMTSL2 Gly656Ser mutant, and further, the impact of the mutant on TGF-β pathways in future studies.

Collectively, our study has noted the clinical phenotype of a female adult patient with geleophysic dysplasia as implicated by her homozygous ADAMTSL2 p. Gly656Ser mutation. Our findings may serve as a reference for clinicians. Further analysis on posttranslational modification and secretion alterations of ADAMTSL2 Gly656Ser mutant will increase our understanding of the pathobiology of geleophysic dysplasia.

## Data Availability

The datasets for this article are not publicly available due to concerns regarding participant/patient anonymity. Requests to access the datasets should be directed to the corresponding author.
