# Supplementary material for: Case report: A homozygous ADAMTSL2 missense variant causes geleophysic dysplasia with high similarity to Weill-Marchesani syndrome
Source: Front Genet. 2022 Sep 28;13:1014188. doi: 10.3389/fgene.2022.1014188 (PMC9554500; doi:10.3389/fgene.2022.1014188)
Supplement: Supplementary file 1 [file DataSheet1.PDF]

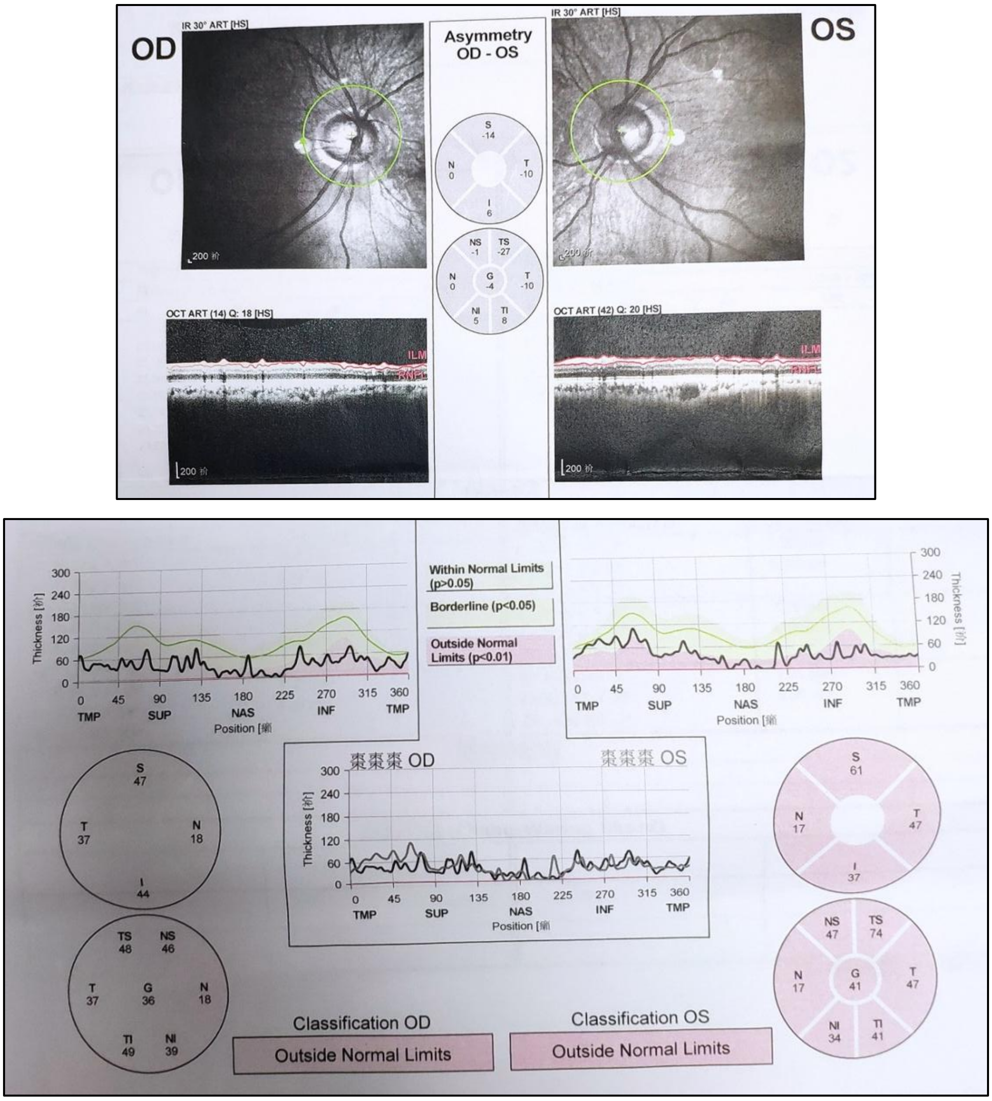

Figure S1 Optical coherence tomography (OCT) revealed reduced retinal nerve fiber layer thickness.

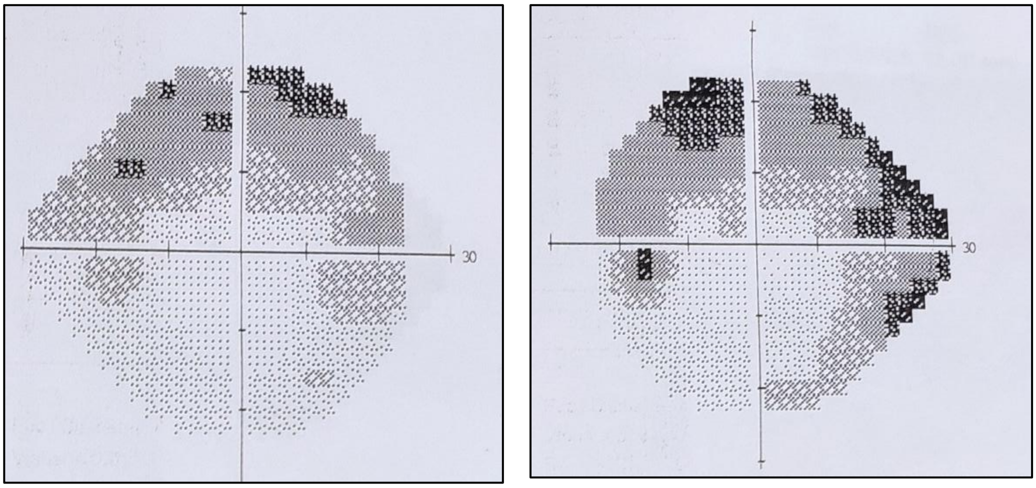

Right eye Left eye

Figure S2 Single field analysis revealed peripheral arcuat visual field defect at upper temporal quadrants of both eyes.

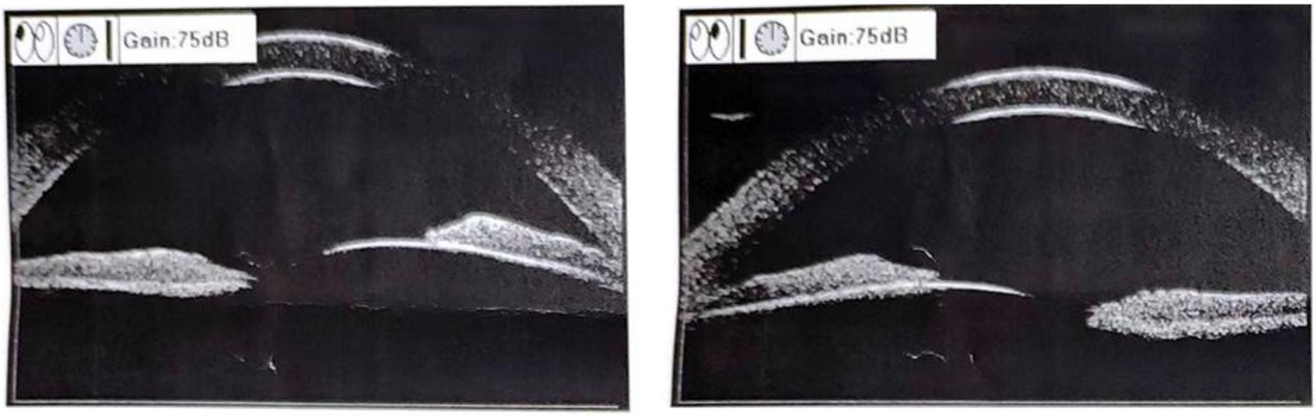

Right eye Left eye

Figure S3 Ultrasound biomicroscopy (UBM) examination revealed shallow anterior chamber and iris pushed forward.
